# Hand, foot and mouth disease: spatiotemporal transmission and climate

**DOI:** 10.1186/1476-072X-10-25

**Published:** 2011-04-05

**Authors:** Jin-feng Wang, Yan-Sha Guo, George Christakos, Wei-Zhong Yang, Yi-Lan Liao, Zhong-Jie Li, Xiao-Zhou Li, Sheng-Jie Lai, Hong-Yan Chen

**Affiliations:** 1 LREIS, Institute of Geographic Sciences and Natural Resources Research, Chinese Academy of Sciences, Beijing 100101, China; 2 Department of Geography, San Diego State University, San Diego, CA 92182-4493, USA; 3 Chinese Center for Disease Control and Prevention, Beijing 102206, China; 4 Institute of Population Research, Peking University, Beijing 100871, China; 5 School of Environmental Sciences, University of Liverpool, Liverpool, UK

## Abstract

**Background:**

The Hand-Foot-Mouth Disease (HFMD) is the most common infectious disease in China, its total incidence being around 500,000 ~1,000,000 cases per year. The composite space-time disease variation is the result of underlining attribute mechanisms that could provide clues about the physiologic and demographic determinants of disease transmission and also guide the appropriate allocation of medical resources to control the disease.

**Methods and Findings:**

HFMD cases were aggregated into 1456 counties and during a period of 11 months. Suspected climate attributes to HFMD were recorded monthly at 674 stations throughout the country and subsequently interpolated within 1456 × 11 cells across space-time (same as the number of HFMD cases) using the Bayesian Maximum Entropy (BME) method while taking into consideration the relevant uncertainty sources. The dimensionalities of the two datasets together with the integrated dataset combining the two previous ones are very high when the topologies of the space-time relationships between cells are taken into account. Using a self-organizing map (SOM) algorithm the dataset dimensionality was effectively reduced into 2 dimensions, while the spatiotemporal attribute structure was maintained. 16 types of spatiotemporal HFMD transmission were identified, and 3-4 high spatial incidence clusters of the HFMD types were found throughout China, which are basically within the scope of the monthly climate (precipitation) types.

**Conclusions:**

HFMD propagates in a composite space-time domain rather than showing a purely spatial and purely temporal variation. There is a clear relationship between HFMD occurrence and climate. HFMD cases are geographically clustered and closely linked to the monthly precipitation types of the region. The occurrence of the former depends on the later.

## Introduction

Hand-Foot-Mouth Disease (HFMD) is the most common gastrointestinal infectious disease in China, mainly among children less than 5 years old, about 91% [1]. HFMD is caused by viruses that belong to the enterovirus genus (group). This group of viruses includes polioviruses, coxsackieviruses, echoviruses, and enteroviruses. The virus transmits through fecal-oral and/or respiratory droplets, or by stool touching, respiratory secretions, herpes solution and polluted staff of patients. The virus can be detected from the stool and pharynx of patients several days before falling ill, the infection reaches the highest point after one week of falling ill, and the stool virus discharges during the last several weeks. Usually the enterovirus exhibits strong transitivity, the latent infection is high and the transmission paths are complicated, causing large epidemics in short times. The disease causes fever, tetter and ulceration on hand, foot and mouth, and may further develop into myocarditis, pulmonary edema, aseptic meningoencephalitis, and other complications [2,3]. The disease has high infection in China: 488,955 reported cases during the year 2008, with morbidity 37/100,000, mortality 0.0095/100,000 and ill-death rate 0.26/1000, and 1,155,525 cases during 2009.

Many studies have been conducted in recent years seeking to understand the HFMD transmission patterns, and to design evidence-based control strategies. For instance, a significant association between weekly HFMD incidence and 1-2 weeks lagged weekly temperature and rainfall was found in Singapore [4]. From 1979 to November 2010 a total number of 2566 papers indexed by the subject of HFMD were published in Chinese journals. About 80% of the total number of the papers was published during the period 2008-2010, whereas the most intense period of the disease occurred during 2005-2010 [5]. The literature generally focuses on the description and assessment of local outbreaks, incidence and prevalence, demographic distribution among professionals, age, sex, urban/rural, seasons, kindergarten/scattered, clinic characteristics, cure, and responsible virus (EV71, CoxA16). These studies have found that infants and children less than 5 years old are commonly susceptible to the virus (children are more likely to be at risk for infection and illness because they are less likely than adults to have antibodies to protect them; such antibodies develop in the body during a person's first exposure to the enteroviruses that cause HFMD). Although a specific preventive for HFMD is not yet available, the infection risk can be generally lowered by following good hygiene practices. Statistically, the relative risk is expressed by the OR (Odds Ratio) or the RR (Relative Risk), which provides a measure of how many times the relative risk of the exposed group is contained in non-exposed group. The significant disease risk factors are: rural/urban areas (OR = 2.1), drinking behavior (OR = 2.441), infants washing hands before dinner (OR = 0.505) [6]; float population (OR = 4.507), toy sucking (OR = 3.220) [7], and low income families [8]. There are certain controversial reports concerning the severity of the disease among kindergarten and scattering children [9,10]. HFMD is closely correlated with population density and communication [10]. Cities with higher population density and increased float population are at increased risk to the disease, the incidence in the buffer zone between urban and rural being much higher than in both the urban and rural areas [11]. The situation is much more severe in urban than in rural regions, and disease prevalence in plain terrain is higher than in mountainous areas. During May and June, the high disease clustering starts moving from South to North China [12]. The controversial reports on the relative risk of HFMD in rural and urban areas might be due to the different grouping of the buffer zones between rural and urban areas. In many cases the disease symptoms are difficult to be identified by regional health services (doctors etc.), which makes HFMD difficult to control.

Important issues that remain unclear include the spatiotemporal pattern of the HFMD outbreaks in China, and what role climate plays in the transmission of the disease in the space-time domain. As has been reported in the relevant literature, CoxA16 strains are broadly distributed geographically, increased incidence of EV71 infection in young children occurred more often in geographic areas with increased mortality rates [13], and the genotypes of EV71-associated HFMD differ in space and time [14,15]. Also, climate indicators can be valuable in the prediction of HFMD activity, which could assist in explaining observed disease peaks across space-time [16]. Answering space-time issues and disease-climate associations can provide valuable information regarding the allocation of public health resources for prevention and treatment purposes [17-19].

Prospective cohort studies could be used, but they are relatively expensive due to the cost of recruiting many individuals who will never be infected, and the high staff cost of the reactive follow-up by medical personnel. A carefully designed prospective cluster study could provide a more efficient way of gathering key data to improve basic understanding of infectious disease transmission dynamics, although substantive problems related to space-time disease change remain unresolved [20]. In fact, most analytical methods used in outbreak detection studies are purely temporal [21-23], which means that these methods can be late at detecting outbreaks that start locally and are linked to serious multiple testing problems generating false signals [24]. Scan statistics methods attempting to resolve such issues are of rather limited usefulness since they make assumptions that are often unrealistic (e.g., a uniform population at risk or ad hoc probability models), or they require information that may be not easily obtainable (e.g., information about the geographical and temporal distribution of populations at risk). Some studies of disease outbreaks (e.g., those based on prospective space-time permutation scan statistics) consider separately purely spatial and purely temporal variations [24,25], which is a simplification of the natural fact that the disease propagates in a composite space-time domain affected by regional climate dynamics. Significant effort has been made by means of the Kulldorf method to improve the ability to find spatial outbreaks using univariate input. This includes our ongoing study to develop a new method to detect multiple clusters in a study area by constructing two or more clusters in the context of the alternative hypothesis. In fact, many of the above methods have not been designed to account for important associations between disease distribution and meteorological conditions

Given the difficulties of previous statistical studies as regards the handling of the high spatiotemporal data dimensionality and the rigorous representation of composite space-time disease variation, in this work we use the space-time BME-S method, which is a combination of the Bayesian Maximum Entropy (BME) theory and the Self-Organized Map (SOM) technique [26]. The BME-S avoids certain modeling simplifications and dimensionality problems of previous studies and offers a realistic framework for modeling and estimation of the disease distribution in a composite space-time domain. Using readily available and well-tested BME-S software, the present HFMD study provides valuable insight into the disease space-time structure and mechanisms in China and their relation to the meteorological attributes and indicators of the region. Otherwise said, the BME-S methodology considers disease propagation and outbreak detection as interdisciplinary problems, which require the integration of information bases from different fields, e.g., health, environmental and population sciences [27,28].

## Methods and Data

### BME

Epidemiologic patterns vary by geographical region and time period. To incorporate these significant space-time characteristics of the disease and their relationship to regional climate dynamics let *X**_p _***denote a spatiotemporal natural attribute in general (HFMD cases, meteorological indicator, disease determinant, incidence etc.), where ***p ***= (***s***,*t*) denotes a space (***s***)-time (*t*) point under conditions of in situ uncertainty. A detailed technical presentation of the BME theory can be found in Christakos [29] and Christakos et al. [30]. Briefly, the BME theory introduces a general knowledge synthesis framework for spatiotemporal modeling and mapping purposes in a manner that incorporates the general (core) knowledge base, *G*-KB (consisting of natural laws such as heredity and mutation, theoretical models such as SIR model, scientific theories such as mass conservation, and empirical relationships such as dose-response elastic) and specificatory knowledge base, *S*-KB (site-specific knowledge like hard data, uncertain information, secondary sources) of the in situ situation. In mathematical terms BME involves the following two basic equations

where *f*_*X*;*k *_is probability density function (pdf) of the attribute *X**_p _***at each space-time point *p_k_*; *A *is a normalization parameter; ***g***_*G *_is a vector with elements representing the *G*-KB; ***μ***_*G *_is a space-time vector with elements that assign proper weights to the elements of ***g***_*G *_and are the solutions of the system of equations and ***ξ***_*S *_represents the *S*-KB available. Unlike previous statistical studies of HFMD, the BME theory can incorporate core knowledge *G *in terms of epidemic laws, when available, in addition to site-specific information *S *in terms of case numbers.

Space-time estimation and mapping methods based on BME automatically include non-linear estimators [31-36]. For example, the mean BME estimates of HFMD cases across space-time are obtained from *f*_*X*;*k *_at each point *p_k _*as follows [26,37]:

The associated estimation error standard deviation is given by

at each point *p_k _*in the space-time domain of interest.

### SOM

**Figure 1:**
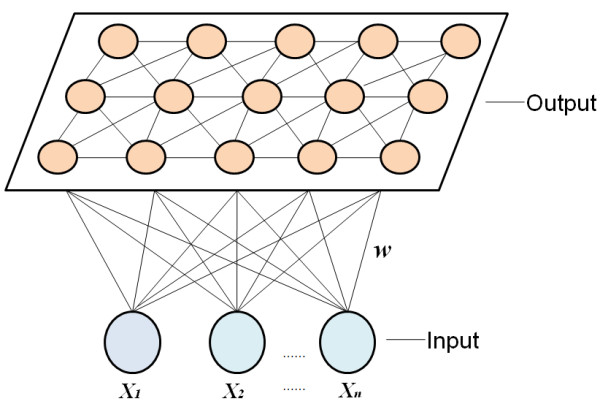
**Structure of SOM**.

SOM is an effective and practical tool for dimensionality reduction [38] that can map individual, *n*-dimensional, data vectors into a low-dimension display space based on the original data structure. SOM includes two layers (Figure 1)[39]: the input and output layers. The input layer simulates the retina of perceiving/receiving external input information, whereas the output layer simulates the responding cortex and its neurons forming a neighborhood structure; the output layer is two-dimensional. The input layer is connected to the relevant weights on the output layer. The former involves training data and the latter consists of neurons to be trained to represent the categories or clusters of the imported data using an algorithm in which the nearby neighbor neurons are encouraged to assume similar values, whereas distant neighbors are mutual suppressed. A more detailed discussion of SOM is given below in terms of the HFMD study.

### BME-S

We studied the relationship between the observable disease variation in the space-time population domain and the underlying transmission mechanisms on the basis of the HFMD data available. SOM techniques were used to reduce the high dimensionality of the spatiotemporal HFMD cases into a two dimensional map that discloses disease dynamics at the country level and to explore the underlining demographic and physiologic determinants. In order to investigate the commonly suspicious role of climatology, the station-based climate observations are interpolated across space-time by means of the BME theory. In this way the climate observations are matched to the county-based disease data, and the high dimensionality of the spatiotemporal climate data is also mapped onto the two dimensional framework as the disease using the SOM technique. The method resulting from the integration of BME and SOM is termed BME-S [26]. The subsequent comparison of the BME-S maps of disease distribution and climate variation unveils important disease-climate associations.

### GeoInformation Mechanism

**Figure 2:**
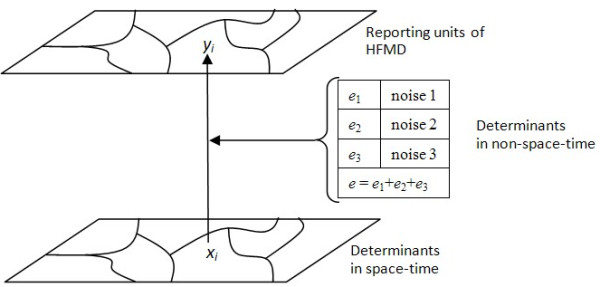
**GeoInformation mechanism**.

Figure 2 illustrates the observed spatiotemporal pattern of HFMD *Y_i _*in reporting units {*r*}, and underlining spatiotemporal *X_i _*and a spatial {*e*} determinants of the disease; the function Θ maps *X_i _*onto *Y_i_*. Potentially high {*e*} non-space-time values can affect spatiotemporal regularity Θ(*X*). The observables *Y_i _*are used to find the underling *X_i _*through Θ, i.e.

every effort has been made to filter out *X *and *e*. In this study, HFMD incidence and climate are set as *X *separately. The BME-S method is used as Θ because it can filter out spatiotemporal regularity (*Y*) of *X *by retaining spatial topology in clustering; BME-S is robust to extreme values by normalization of input.

### Data

**Figure 3:**
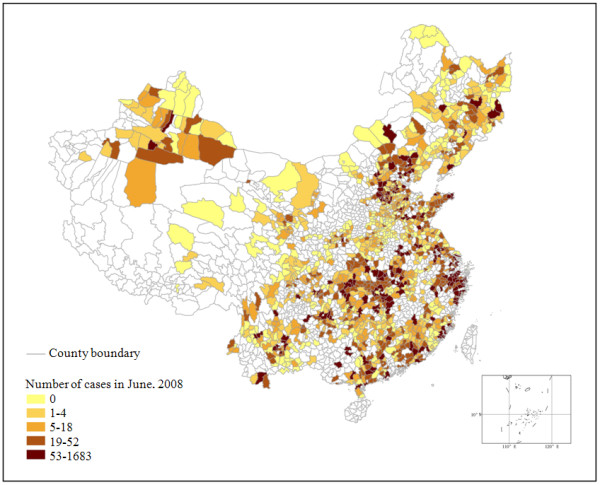
**Number of HFMD cases in Jun 2008**.

Two spatiotemporal datasets were used in this study: The first is the monthly number of HFMD cases from the May 1 2008 to March 27 2009, see Figure 3, for example. This dataset was obtained from the Chinese Center for Disease Control and Prevention (CDC). Due to very sparse inhabitance of the west part of China, we focused on the east part of the country, where the number of disease cases is available at 1456 counties during 11 months. The second dataset consists of the meteorological observations during the disease period, and was downloaded from the website of China Meteorological Data Sharing Service System. This dataset includes 11 meteorological indicators measured at 674 stations during each month. These indicators are the average air pressure, average temperature, average maximum temperature, average minimum temperature, average humidity, average wind speed (these six indicators are the averages of daily records during each month), maximum and minimum temperatures (the maximum and minimum observed during each month), temperature difference (difference between the average maximum and minimum temperature during each month), precipitation and sunshine hours (accumulated records during each month). All the 11 meteorological indicators are included into the analysis in order to find which of them are significantly associated with HFMD transmission. The meteorological records in 11 months and 674 stations were interpolated into 1456 counties by using the BME method at the space-time resolution of the HFMD case data. Both the spatiotemporal HFMD incidences and each of the climate indicators are organized into a matrix composed by 1456 rows (counties) and 11 columns (months) respectively, or 1456 × 11 cells, or 1456 sample unit vectors, each of the sample units is a vector with 11 dimensions, then the cells are normalized and the sample units are ready for input into SOM algorithm.

The SOM algorithm used in the HFMD study involves the following steps: (1) Select the topology of the output layer, which consists of a number of neurons, represented as "rectangle" or "hexagon", and arranged one by one; the number of the neurons corresponds to the number of categories to be classified (4 × 4 or 50 × 50 categories in this study); (2) Initialization: each of the neurons is weighted by a vector of small random values, ranged between 0 and 1, and the vector dimension is equal to the number of variables of a sample unit in the input layer, i.e., 11 (months) in this study; (3) Compute the Euclidean distances between the 1456 samples (unit vectors) and the weight vectors associated with each output neuron as follows , where *X_k _*= [*X*_1*k*_,..., *X_nk_*] is a *k*-th input sample unit vector, *i *is the number of variables or dimensions of each input sample unit vector, same as the dimension of a output neuron vector (*i *= 1, ..., 11 in this study), and *j *= 1, 2, ..., *p*, where *p *is the number of neurons in the output layer, i.e. 4 × 4 or 50 × 50 in the present study. (4) Determine the winning neuron, that is the one with the minimum distance among all output neurons *j*, giving an input sample unit vector *k*; (5) Compute the neighborhood of the winning neuron, generally, which is a rectangle or circle with this neuron as the center. The initial neighbor include all the output neurons, then increase the iteration time and the neighborhood range decrease, at last, the range only include the winning one; (6) Adjust the weights contained in the neighborhood of the winning neuron *j *by the following formula,

where *α*(*t*) > 0 denotes the learning rate. (7) Start the next iteration from step (1) until a predefined time is reached; usually the larger the *t *is, the more stratified are the neurons [40,41].

### Normalization

A dataset is classified by SOM into a number of categories according to data similarities. Although similarities could be detected either in the magnitude or in the structure of two vectors, the later is the focus of the present HFMD study. In order to study (a) the overall spatiotemporal indicator similarity, (b) the temporal similarity of spatial patterns, and (c) the spatial similarity of temporal patterns, we need to remove the magnitude differences between all 1456 × 11 spatiotemporal cells, between the 11 time slices, and between the 1456 counties, respectively. Both the HFMD incidence and the climate indicators are re-scaled within the 0-1 range.

Three kinds of normalizations were conducted: (i) global normalization in which all values were normalized into the 0-1 range based on all 1456 × 11 spatiotemporal cells; (ii) time normalization in which all values were normalized into the 0-1 range among the 1456 counties for each of the 11 time slices, so that the between months' difference of the HFMD magnitude is removed and the similarity of the spatial pattern between months is focused; (iii) cell normalization in which all values were normalized within the 0-1 range among all 11 months for each of the 1456 counties, so that the between counties' differences of the HFMD incidence or a climate indicator are reduced and the focus is the similarity of temporal patterns between counties.

Normalization removes the attribute magnitude effect to allow the comparison between spatiotemporal, spatial, and temporal structures. It would be helpful to keep in mind that the spatiotemporal type is a second order characteristic, i.e., the spatiotemporal structure of a quantity rather than the quantity itself.

## Results and Interpretation

### HFMD-Climate Associations

**Figure 4:**
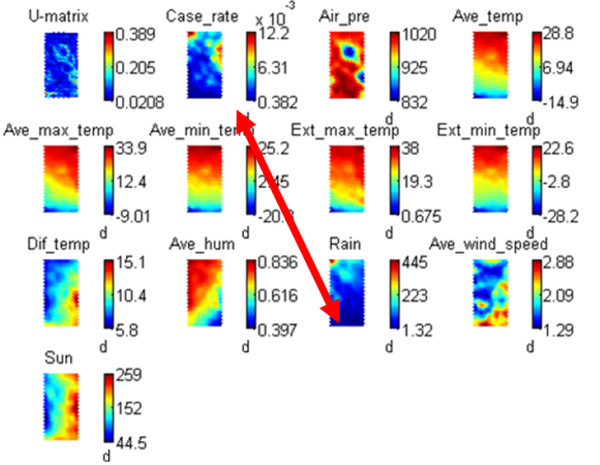
**Component planes for HFMD and meteorological indicators**.

We explored the relationship between HFMD and the relevant climate indicators by studying the similarity of their spatiotemporal patterns. The SOM is predefined to include 50 × 50 neurons and the output is shown Figure 4. U-matrix visualizes the distances between the neighboring map units, and serves as a baseline for checking for possible spatiotemporal input clustering. In the U-matrix there are more units than the predefined map size; for example, in the case of [m_1_, m_2_, m_3_], i.e. a 1 × 3-sized map, the U-matrix is a [1 × 5] vector [u_1_, u_12_, u_2_, u_23_, u_3_], where u_ij _is the distance between i and j, and u_i _is the average distance between the current unit i to its neighboring units [42].

Figure 4 shows that the planes related to temperatures including average temperature (Ave_temp), average maximum temperature (Ave_max_temp), average minimum temperature (Ave_min_temp), extreme maximum temperature (Ext_man_temp) and extreme minimum temperature (Ext_min_temp) are very similar, in which case we used TEMP to generally denote the temperature-related quantities in the present study. Temperature differences (Dif_temp) and sunshine hours (Sun) are somewhat similar, but are negatively correlated to the average humidity (Ave_hum) and rainfall (Rain). The HFMD incidence SOM (Case_rate) is to some extent correlated to TEMP SOM, and is quite similar to the Rain SOM, which means that the spatiotemporal transmission of HFMD is closely related to the spatiotemporal pattern of rainfall or its confounding factor(s). Below we will discuss the association in more detail.

### HFMD Spatiotemporal Types

**Figure 5:**
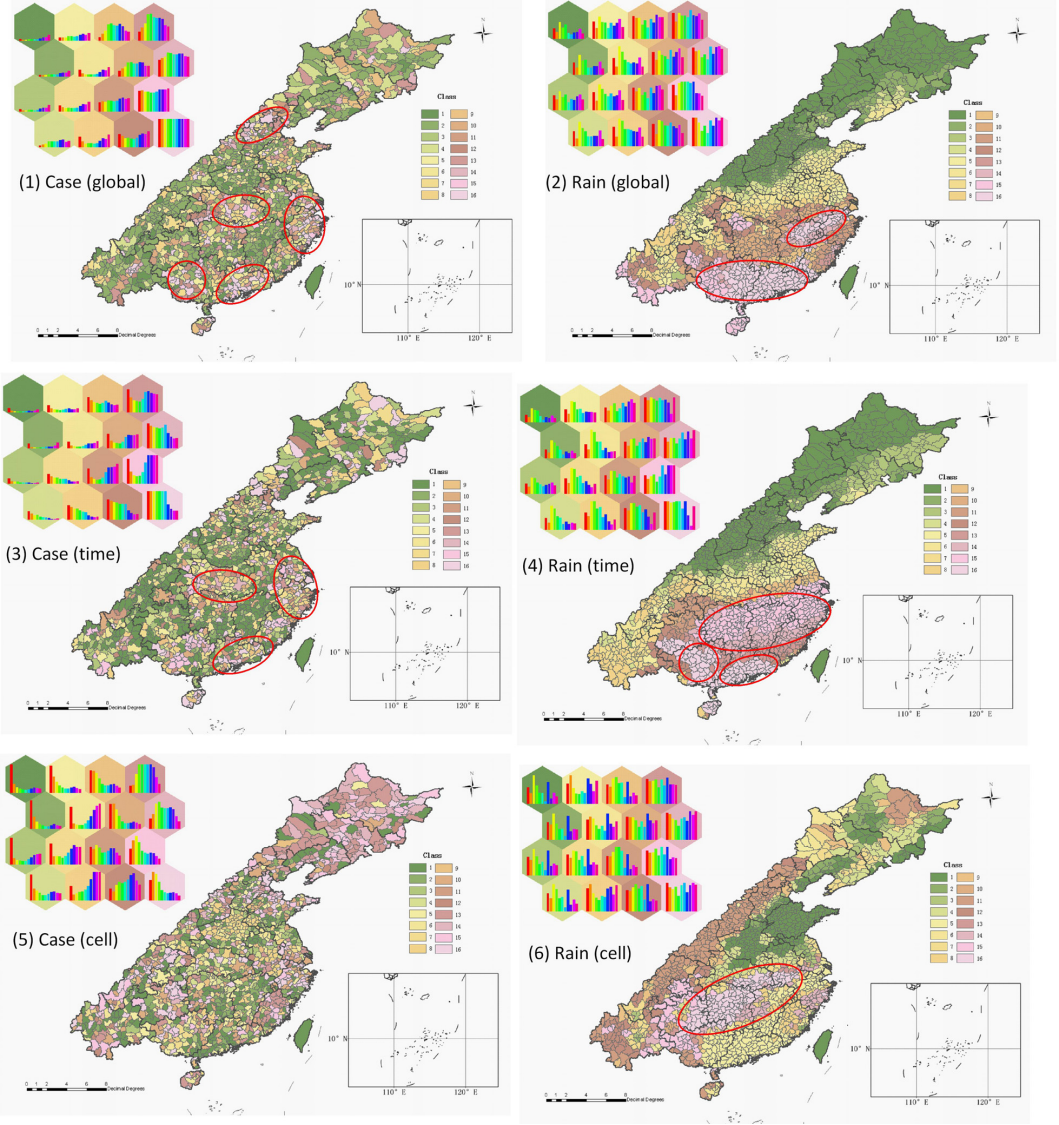
**Spatiotemporal types and inside time series of (1) global normalized HFMD incidence; (2) global normalized month precipitation; (3) time normalized HFMD incidence; (4) time normalized month precipitation; (5) cell normalized HFMD incidence; (6) cell normalized month precipitation**.

Disease and climate indicators are classified by SOM into 16 types (Figure 5). The number 16 is predefined in this study (the bigger the number, the finer the classification). The study area is mapped onto the SOM space. Each county corresponds to one of the 16 best-matching neurons on the SOM space, marked by one color. Colors from "green" to "pink" denote an increasing level of HFMD incidence in Figure 5(1, 3, 5), and an increasing level of Rain in Figure 5(2, 4, 6). The monthly series of HFMD incidence and Rain during the 11 months of the study are presented by 11 bars within each hexagon representing the time structure of the relevant attributes in the spatiotemporal types. The monthly series are less varied in Figure 5(1), whereas they show higher variation in Figure 5(3, 5).

To readily understand and interpret the spatiotemporal type, below we start with the precipitation Figure 5(2, 4, 6) using existing knowledge on climate; then, we proceed with the disease Figure 5(1, 3, 5); and finally Figure 5(1, 3, 5) are compared with Figure 5(2, 4, 6). More specifically, Figure 5(2, 4, 6) show that the spatiotemporal precipitation types are spatially compact, i.e., the precipitation pattern is continuous across space-time. However, this is not the case with HFMD incidence, Figure 5(1, 3, 5), where the corresponding spatiotemporal types are much less spatially continuous. Figure 5(2) shows greater similarity to Figure 5(4) than to Figure 5(6), which means that the global spatiotemporal types Figure 5(2) are controlled mainly by the similarity of spatial patterns rather than by the similarity of temporal series. At a global level, there is an obvious northeast to southwest belt in Figure 5(6), which is completely consistent with the country's large scale geomorphic and the southeast seasonal wind (both have maximum variation along the northwest-southeast direction). Within the southeast seasonal wind domain, Figure 5(4) shows a greater variability than Figure 5(6), because the spatial precipitation pattern (Figure 5(4)) is more influenced by the local geomorphic than by the monthly precipitation series (Figure 5(6)). There is a big tongue extending into the country along the Yangtze river basin in Figure 5(2), which reflects the spatial similarity of the spatiotemporal precipitation structure in the region.

Just as is the case with the similarity of Figure 5(2, 4), the overall HFMD spatiotemporal type Figure 5(1) is controlled mainly by the spatial disease pattern (Figure 5(3)) rather than by the temporal pattern (Figure 5(5)). Unlike the monthly precipitation Figure 5(2, 4, 6), the Figure 5(1, 3, 5) of the HFMD spatiotemporal types are highly varied throughout the country and less spatially continuous (i.e., characterized more by local outbreaks). There are four clear spatial clusters of high HFMD incidence: Yangtze delta, Middle china, Peal delta and the neighbor to Vietnam in Figure 5(1, 3). The clusters are marked by circles and seem to be spatially consistent with the precipitation spatiotemporal clusters in Figure 5(2, 4). The above clearly imply that the HFMD outbreaks are affected by the spatiotemporal types of monthly precipitation. Besides spatial clusters, the Figure 5(1, 3, 5) illustrates the existence of first case occurrences across the country, i.e. HFMD cases occurring in villages, communities, kindergartens, schools or counties that are epidemiologically independent in space and time (this sporadic feature cannot be explained in terms of climate).

### Spatialization-combined factors

**Figure 6:**
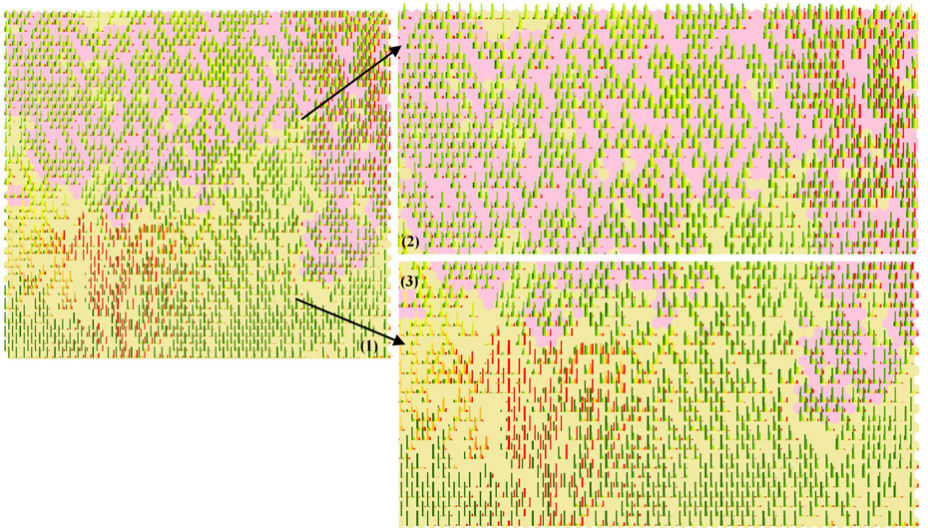
**SOM clusters: (1) Overall graph; (2) Enlarged graph of pink part (precipitation); (3) Enlarged graph of yellow part (HFMD incidence)**.

Next, we explored the similarity of HFMD spatiotemporal type and precipitation. The dataset was organized into 2912 rows (HFMD incidence and one factor, each having 1456 rows) and 11 columns (months), and then classified in terms of a 50 × 50 SOM. The best-matching neuron in SOM for each cell in the geographic map is found and assigned a color for each attribute (histograms only for the best-matching neurons of each factor are presented). The output Figure 6(1) is the overall graph, and the Figure 6(2, 3) are enlarged parts corresponding to the two attributes. If HFMD and precipitation were completely irrelevant, we would expect that the two attributes are completely separated in the SOM map. However, some HFMD clusters (yellow color in Figure 6) are within precipitation cluster (pink in Figure 6), implying that in some parts of the country the HFMD outbreak are associated with certain spatiotemporal precipitation types, although the later might not constitute an efficient condition as indicated by the remaining parts of Figure 6 (the two attributes are separated into contiguous regions). These conclusions are consistent with the findings in Figure 5(1, 3) and Figure 5(2, 4), where HFMD spatiotemporal outbreaks were shown to be within the precipitation spatiotemporal clusters.

The peak time varies among the different regions of the country. HFMD incidence is relative high in many parts of the country during May-July 2008. Peak periods in regions with higher precipitation levels also occurred during May-August, and then during May 2008 (this is clearly shown in Figure 6), which verified the existence of a certain incidence-precipitation association.

## Conclusion and Discussion

The present study investigated composite space-time distribution of HFMD cases and their relationship with regional climate indicators. The spatiotemporal datasets with different formats were matched by the BME theory, and the high dimensionality datasets (in which the spatial and temporal topology was retained) were reduced and mapped onto two-dimensional maps. 16 spatiotemporal types of HFMD cases and climate indicators were identified in these maps, and an association between them was detected.

Besides the significant association between HFMD spatiotemporal type and monthly precipitation spatiotemporal type, we also applied data exploratory analysis and the BME-S method to other climate indicators and we found the next to the significant factors. The pressure distribution during the 11 months (maps not shown here) was relatively stable in most regions. During May-August 2008 the pressure was a little low, then it rapidly rose until its maximum in January 2009; after that the pressure began declining, which was the opposite behavior from that of the incidence distribution. Temperature variation (maps not shown here) was the opposite behavior from that of pressure variation, and seems to suggest a certain similarity between temperature and HFMD variation.

The association between the HFMD spatiotemporal clusters and the spatiotemporal types of monthly precipitation makes it possible to forecast the risk of a disease outbreak on the basis of the prediction of spatiotemporal precipitation types (obtained by means of atmospheric science methods). Intervention and prevention measures should focus predominantly on kindergartens and junior schools located in the HFMD risk areas during the risk periods estimated by the physical methods of atmospheric science and meteorological forecasting.

Compared to similar studies on HFMD (i.e. demographic distribution among professionals, age, sex, urban/rural, seasons, kindergarten/scattered, clinic characteristics, cure, virus and time series association between HFMD and climate), this study identified the spatiotemporal types of HFMD, and its association with precipitation in a large territory. An advantage of the study is that it takes advantage of the fact that we adopt the recently proposed methodology (BME-S) to analyze efficiently the relatively large volume of multi-dimensional data, where the complexity stems from processing several indicators and the disease data in the three-dimensional space-time continuum. Essentially, BME-S is a combination of the BME technique for geostatistical space-time prediction of the indicators' values at unsampled locations in the study area, and of the SOM technique to handle and map efficiently multi-dimensional information. BME does not suffer certain well-known drawbacks of mainstream statistical estimation techniques such as Kriging [43]: like other methods of the statistical regression type [44], Kriging is restricted to the first-and second-order spatial moments of the attribute, it is a linear interpolator that relies on the Gaussian assumption [45], and it uses mainly hard (i.e., exact) or hardened data available at a set of neighboring points [46]. SOM creates a topologically ordered partition in a visible two-dimensional plane. In other words, the first cluster is near to the second cluster but away from the ninth cluster. The relationship among the clusters is indicated by means of the order and is clearly displayed on the plane. The topology of the SOM helps it outperform mature methods such as hierarchical, k-means [42,46]. There are two criteria to evaluate the created SOM: the first one is data representation accuracy, and the other one is the accuracy of the dataset topology considered.

The limitation of the study is that we used 11 months' data (rather than data from the whole year), due to data accessibility issue. The current conclusion is based on monthly data, whereas more accurate findings would have emerged if weekly or daily data were used. HFMD is associated to climate through the interaction between enterovirus activity and human exposure, which both increase during climate change. The biological relationship between climate indicators and enterovius activity is quite complicated, and a mathematical modelling of HFMD spatiotemporal transmission over large territory would display scenarios under different climate conditions. All the above cases deserve to be investigated in future studies.

## Competing interests

The authors declare that they have no competing interests.

## Authors' contributions

JFW provided the idea of the paper, analyzed the relationship between HFMD and meteorological factors, and drafted the manuscript. YSG was mainly responsible for processing the data and debugging the programs. GC instructed the realization of BME and revised the manuscript. WZY participated in the analysis between HFMD and meteorological factors. YLL participated in selecting the potential meteorological factors. ZJL participated in the analysis between HFMD and meteorological factors. XZL and SJL collected and rearranged the original data. HYC made some revision for the manuscript. All authors read and approved the final manuscript.

## Supporting Information

The space-time BME and SOM software used in this paper is available, free of charge, via the websites http://homepage.ntu.edu.tw/~hlyu/software/SEKSGUI/SEKSHome.html, with paper [47], and http://www.cis.hut.fi/somtoolbox/download/, with paper [42,48].
